# Tomographic and Electron Microscopy Description of Two Bone-Substitute Xenografts for the Preservation of Dental Alveoli

**DOI:** 10.3390/ijms252010942

**Published:** 2024-10-11

**Authors:** Lemy Vanessa Barba-Rosado, Maria-Fernanda Realpe, Carlos-Humberto Valencia-Llano, Diego López-Tenorio, Ismael Enrique Piñeres-Ariza, Carlos David Grande-Tovar

**Affiliations:** 1 Grupo de Investigación en Fotoquímica y Fotobiología, Programa de Química, Facultad de Ciencias Básicas, Universidad del Atlántico, Puerto Colombia 081008, Colombia; lbarba@mail.uniatlantico.edu.co; 2 Grupo Biomateriales Dentales, Escuela de Odontología, Universidad del Valle, Calle 4B # 36-00, Cali 760001, Colombia; maria.fernanda.realpe@correounivalle.edu.co (M.-F.R.); carlos.humberto.valencia@correounivalle.edu.co (C.-H.V.-L.); diego.lopez.tenorio@correounivalle.edu.co (D.L.-T.); 3 Grupo de Investigación Física de Materiales, Programa de Física, Facultad de Ciencias Básicas, Universidad del Atlántico, Puerto Colombia 081008, Colombia; ismaelpineres@mail.uniatlantico.edu.co

**Keywords:** bone substitutes, dental alveolus, tomography, xenografts

## Abstract

After tooth extraction, bone levels in the alveoli decrease. Using a bone substitute can help minimize this bone loss. The substitute can be sourced from a human or animal donor or synthetically prepared. In this study, we aimed to address the following PICOS question: In patients needing dental alveolar preservation for implant placement, how does alveolar preservation using a bovine hydroxyapatite bone xenograft with collagen compare to a xenograft without collagen in terms of changes in alveolar height and width, bone density, and the characteristics of the bone tissue observed in biopsies taken at 6 months? We evaluated two xenograft-type bone substitutes for preserving post-extraction dental sockets using tomography and microscopy to answer that question. A total of 18 dental alveoli were studied: 11 preserved with a xenograft composed of apatite (InterOss) and 7 with a xenograft composed of apatite–collagen (InterOss Collagen). Tomographic controls were performed at 1 and 6 months, and microscopic studies were performed on 13 samples. The biopsies were examined with scanning electron microscopy (SEM) and energy-dispersive spectroscopy (EDS). A Multivariate Analysis of Variance (MANOVA) was conducted in the statistical analysis, revealing a significant increase in bone density over time (*p* = 0.04). Specifically, bone density increased from an average of 526.14 HU at 30 days to 721.96 HU at 60 days in collagen-free samples. However, no statistically significant differences in height or width were found between groups. The MANOVA results indicated that the overall model had a low predictive ability for height, width, and density variables (R-squared values were low), likely due to sample size limitations and the complexity of bone tissue dynamics. On the other hand, FTIR analysis revealed the presence of phosphate groups, carbonates, and amides I, II, and III, indicative of inorganic (hydroxyapatite) and organic (type I collagen) materials in the xenografts. TGA and DSC showed high thermal stability, with minimal mass loss below 150 °C. Finally, both xenografts were influential in alveolar bone regeneration after extraction without significant differences. The trend of increasing collagen density suggests an effect that requires further investigation. However, it is recommended that the sample size be increased to enhance the validity of the results.

## 1. Introduction

After tooth extraction, the bone levels in sockets usually decrease in height and width. To prevent this, preservation techniques involving grafting bone substitutes into the alveoli are used. These techniques can reduce bone resorption by 15% to 50% [1,2].

Among the xenografts available are deproteinized xenografts, which are composed only of mineral-phase tissue (apatite), and those in which collagen has been preserved (or supplemented) to improve biological properties [3,4,5].

Several studies have compared biopsies of human dental sockets grafted with xenografts composed of apatite and xenografts supplemented with collagen using tomographic and histological analysis. For example, Nart et al. conducted a clinical trial comparing dimensional changes and histological composition after using two materials: deproteinized bovine bone mineral (DBBM) and deproteinized bovine bone mineral with 10% collagen (DBBM-C). The study found no statistically significant differences between the two groups in terms of bone level preservation and the composition of newly formed bone and connective tissue [6]. There is also the work of Guarnieri et al. comparing bovine xenografts with porcine xenografts in terms of the resorption of the grafted material [7]. Elizalde-Mota et al. report non-resorbing particles embedded in neoformed bone [8], and Kivovics et al. also report the presence of particles incorporated in the forming tissue [9]. All these studies have histological descriptions in common, and report the presence of remnant particles of the grafted material incorporated in the neoformed bone tissue. In some cases, the particles are integrated without evidence of a fibrous capsule surrounding them. Still, others report the presence of a foreign body reaction, although without evidence of moderate or severe inflammation.

In our previous research, our group successfully implanted two types of xenografts in a murine model. We used histological, histochemical, and SEM techniques to confirm that these xenografts served as scaffolds, promoting the formation of bone tissue in critical-size defects [10]. When reviewing articles that used histological techniques, we noticed that the images closely resemble those in our previous work, depicting bone tissues in formation with varying amounts of unabsorbed xenograft particles [5,11].

However, these studies did not determine which type of xenogeneic bone graft, composed of bovine hydroxyapatite with collagen or without collagen, is most effective in preserving the dental alveolus in patients requiring this intervention for implant placement. Therefore, this research posed the following PICO question as a methodological guide: “In patients who require dental alveolar preservation for implant placement (P), how does alveolar preservation using a bone xenograft composed of bovine hydroxyapatite with collagen (I) compare with a xenograft without collagen (C) in terms of changes in alveolar height and width, bone density, and bone tissue characteristics seen in biopsies taken at 6 months (O)?”

The PICO question allows us to formulate a null hypothesis. When comparing the results between the two groups of alveoli, with and without collagen, no significant differences will be found in the following response variables: height, width, density, and biological characteristics of the grafted areas.

The current study aimed to use SEM/EDS to examine biopsies from areas where xenografts were implanted. This technique allows for detailed high-magnification analysis and chemical examination, enhancing the research findings and helping us better understand the creeping substitution process. Our work reveals differences in the quality of newly formed tissue potentially associated with the presence of collagen. It seems that collagen plays a role in promoting bone growth, highlighting the significance of our results.

## 2. Results

This study presents the findings of grafting dental sockets with xenograft bone substitutes with and without added collagen. We used apatite and apatite–collagen bone xenografts, both made by Sigma Graft (Fullerton, CA, USA). Biopsies of the grafted sockets were taken after 6 months. Clinically, the tissue in the area showed strong integration with the surrounding bone, with very little remaining graft material. SEM analysis confirmed the formation of new tissue and bone cells. The surface of these grafts was analyzed using infrared spectroscopy to study the presence of organic-matter-related functional groups, and their thermal stability was investigated using thermogravimetry (TGA) and differential scanning calorimetry (DSC).

### 2.1. Surgical Procedure and Tomographic Findings in Alveolar Bone Grafts

**Figure 1:**
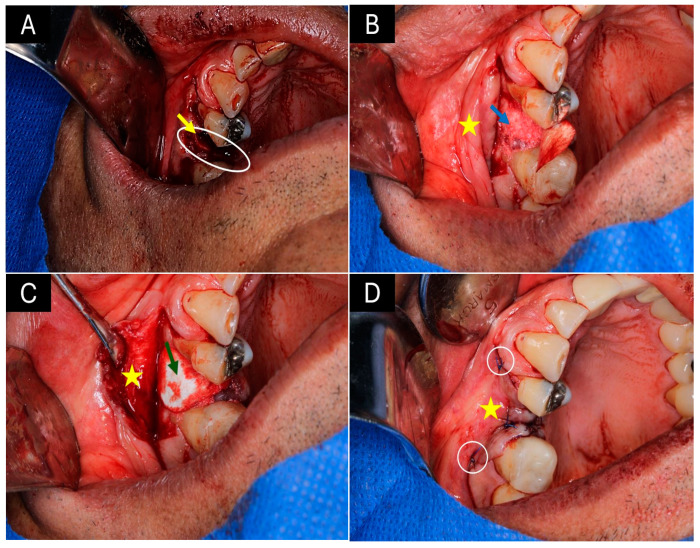
Surgical procedure to preserve the dental socket. (**A**) Post-extraction alveolus area. (**B**) Xenograft placement in the dental socket. (**C**) Placement of the barrier (membrane) protecting the graft. (**D**) Repositioned soft tissues in the grafted area. White oval: Area of the dental socket. Yellow arrow: An area of bone loss on the vestibular surface of the bony ridge. Yellow star: Mucous tissue on the vestibular surface of the bony ridge. Blue arrow: Socket grafted with xenograft material. Green arrow: Collagen membrane in position. White circles: Areas of suture of the tissues.

Figure 1 shows the surgical procedures for applying bone grafts in the dental sockets after extraction. In Figure 1A, the white oval shows the area of the alveolus corresponding to a second upper premolar that has been removed. The yellow arrow indicates a loss of height in the vestibular table of the bony ridge. In Figure 1B, the mucosal tissue lining the bony ridge has been separated on the vestibular surface (yellow star) to expose the socket and place the bone graft. The blue arrow indicates the graft material that has filled both the socket of the dental socket and the area of vestibular bone loss.

In Figure 1C, the green arrow highlights a collagen membrane placed on the surface to protect the grafted area. In Figure 1D, you can see that the mucous tissue has been repositioned and sutured to the rest of the soft tissue.

**Figure 2:**
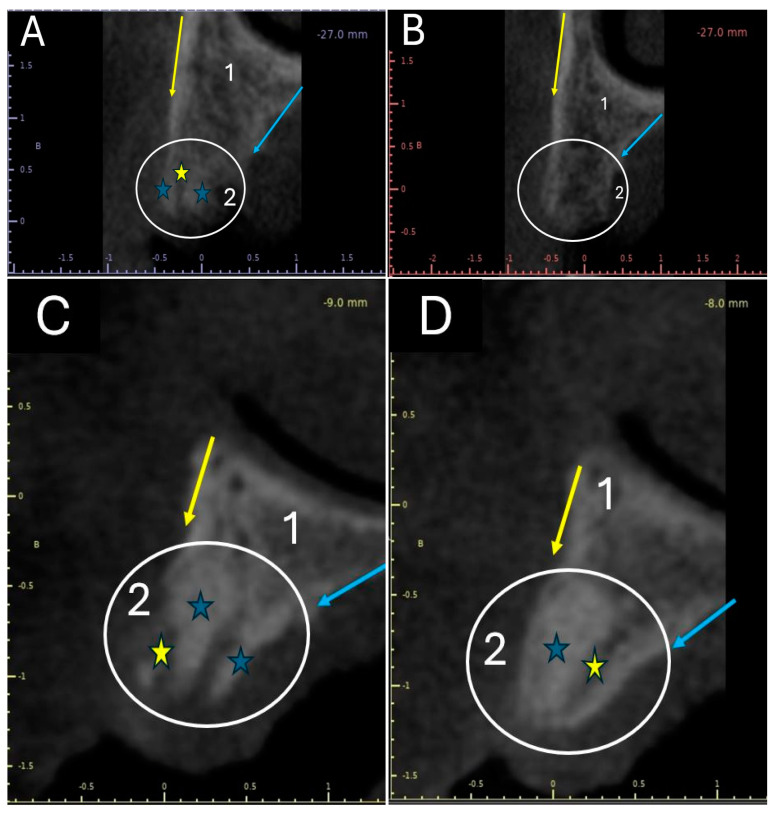
Tomographic images of grafted alveoli in cross-sections. Yellow arrow: The vestibular surface of the ridge. Blue arrow: The palatal surface of the flange. **1**: Area of the alveolus made up of remnant or native bone. **2:** Area of the socket where the graft was placed. Blue stars: Radiopaque appearance zone. Yellow star: Area of radiolucent appearance. White circle: Grafted area of the alveolus. (**A**) Alveolus grafted with apatite–collagen at 1 month. (**B**) Alveolus grafted with apatite–collagen at 1 month. (**C**) Alveolus grafted with apatite at 1 month. (**D**) Alveolus grafted with apatite at 6 months.

Once the grafts were placed, tomographic studies of the grafted alveoli were performed. Figure 2 shows the tomographic images of an alveolus grafted with apatite and another grafted with apatite–collagen, obtained 30 and 180 days post-graft.

Figure 2A shows the image of an alveolus one month after being grafted with apatite–collagen. The circle indicates the area that was preserved with the xenograft. When comparing the part of the alveolus that contains native bone, identified with the number 1, with the part of the alveolus that contains the grafted tissue, identified with the number 2, an area of more radiopaque appearance (blue stars) and a radiolucent area (yellow star) are observed in the grafted area. However, at 6 months, the grafted area (white circle in Figure 2B) presents the radiographic appearance typical of a cancellous bone, with radiopaque and radiolucent areas being very similar to what is observed in the portion of the socket that contains the remaining bone.

Figure 2C shows an apatite-grafted alveolus one month later. The white circle corresponds to the grafted area. Very dense radiopaque areas (blue stars) and some radiolucent areas (yellow stars) can be seen. When comparing sectors 1 and 2, it is observed that the grafted area (identified with the number 2) has more radiopaque or dense areas than the native bone area, identified with the number 1. Figure 2D shows the same socket at 6 months of grafting. The persistence of a very dense radiopaque zone occupying most of the grafted area (blue star in the white circle) is observed, with a radiolucent zone identified with a yellow star. When comparing the area with remnant bone (identified with the number 1) with the grafted area (identified with the number 2), it is observed that zone 1 has a radiographic appearance typical of a cancellous bone. In contrast, zone No. 2 has a very radiopaque appearance.

The radiographic findings in the tomographic images shown in Figure 2 indicate that the alveolus grafted with apatite material presents a slower replacement process than the alveolus grafted with the apatite–collagen material. This suggests that the two materials’ creeping substitution process differs, occurring more quickly with apatite–collagen. This is evidenced in Figure 2B, where, after 6 months, the material appears to have been replaced by spongy-type bone tissue.

**Figure 3:**
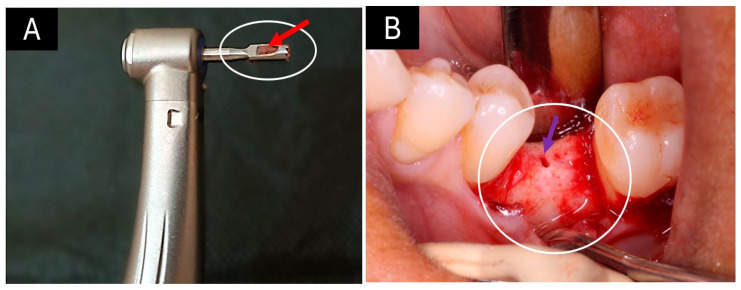
Taking the biopsy. (**A**) Trephine drill bit with a bone fragment inside. (**B**) Area of the ridge where the biopsy was taken. White oval: Trephine drill bit. Red arrow: Bone biopsy inside the trephine. White circle: Bone rim. Purple arrow: A remnant cavity where the biopsy was taken.

Once the 6 months of grafting were completed, a biopsy was taken, as shown in Figure 3. 

A trephine-type bone drill bit was used to take the biopsy, which, being hollow inside, allowed a bone fragment to be taken. Figure 3A shows an image of the trephine (highlighted with a white oval) containing the bone fragment (red arrow). In Figure 3B, the oval corresponds to the area where the socket was grafted after the extraction. The purple arrow points to the cavity left after the biopsy was taken. It is observed that the bone ridge has a smooth, homogeneous, and compact surface without the presence of remaining particles of the grafted material, which indicates that the creeping substitution process has probably already been completed on this surface. This clinical image was similar for all the alveoli operated on, regardless of the type of graft material used.

After the biopsy was taken, the remaining cavity was used by the surgeon in charge of the patient as a guide to complete the surgical preparation necessary to place a dental implant at the site.

### 2.2. Characterization of Apatite and Apatite–Collagen after Grafting

#### 2.2.1. Fourier Transform Spectroscopy (FT-IR)

The FT-IR analysis performed on both the apatite material and the apatite–collagen material after the grafting period showed similar spectra that reveal the presence of the main functional groups belonging to hydroxyapatite (HA) and proteins such as collagen. Figure 1 identifies peaks of 874 cm^−1^, 962 cm^−1^, and 1412 cm^−1^ corresponding to carbonate groups (${CO}_{3}^{2-}$) of HA. Peaks are also observed at 562 cm^−1^, 602 cm^−1^, and 1020 cm^−1^, attributed to orthophosphate groups ${(PO}_{4}^{3-})$ [12,13]. In addition, bands are detected at 3278 and 3076–3078 cm^−1^ associated with the stretching vibrations of the N-H bond in amide I, and a peak of 2933 cm^−1^ due to the stretching vibrations of the group CH_2_. The peak at 1637 cm^−1^ corresponds to the stretching vibration of the C=O bond (amide I), while the bands at 1541 cm^−1^ for apatite and 1543 cm^−1^ for apatite–collagen are attributed to the N-H bending vibration of amide II [14,15]. Likewise, bands are observed approximately in the range of 1200–1240 cm^−1^ for the apatite material and 1204–1237 cm^−1^ for the apatite–collagen material, related to the N-H and C-N bending vibration of amide III. The band at 1450 cm^−1^ is attributed to the C-H bond that indicates the helical structure of type I collagen [16,17]. These characteristic bands of amide groups in the spectra of both xenografts reflect the presence of proteins (mainly type I collagen) deposited in the materials, produced by the interaction with bone tissue [18,19].

**Figure 4:**
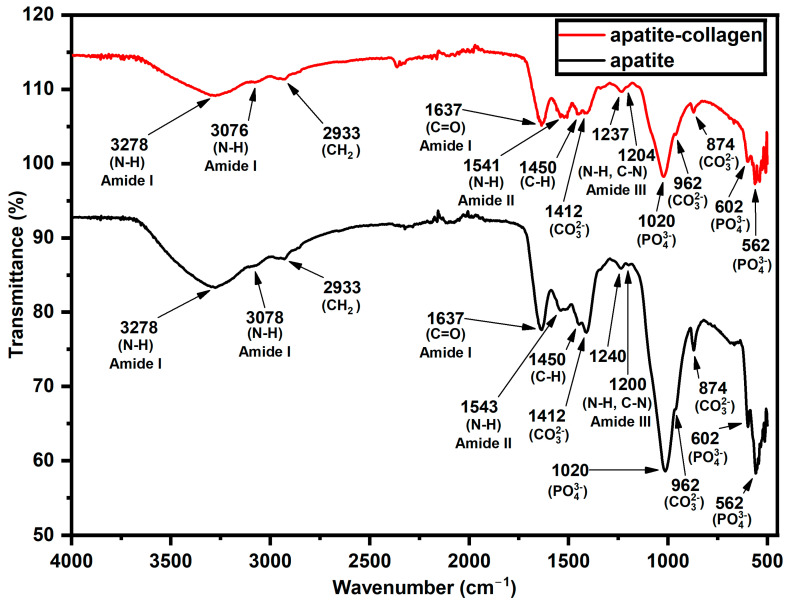
FTIR spectra of apatite (black line) and apatite–collagen (red line).

The functional groups in each spectrum reflect the integration and compatibility of the xenografts with bone tissue [12,15]. The appearance of new bands in the non-collagen (apatite) and collagen-containing (apatite–collagen) materials could be due to the formation of new protein bonds or structures resulting from biological interaction with the extracellular bone matrix [19]. Due to this interaction with the xenografts, an organic bone matrix and an inorganic bone matrix are produced [20]. The organic bone matrix is mainly made up of type I collagen, which, as shown in Figure 4, has amide I, II, and III functional groups. On the other hand, the inorganic bone matrix, the main component of the extracellular bone matrix, comprises hydroxyapatite, which contains carbonates and phosphate functional groups [21,22]. The presence of these functional groups in the spectra of the apatite and apatite–collagen materials confirms the formation of bone tissue.

#### 2.2.2. Thermal Analysis of Xenografts after Grafting Process

**Figure 5:**
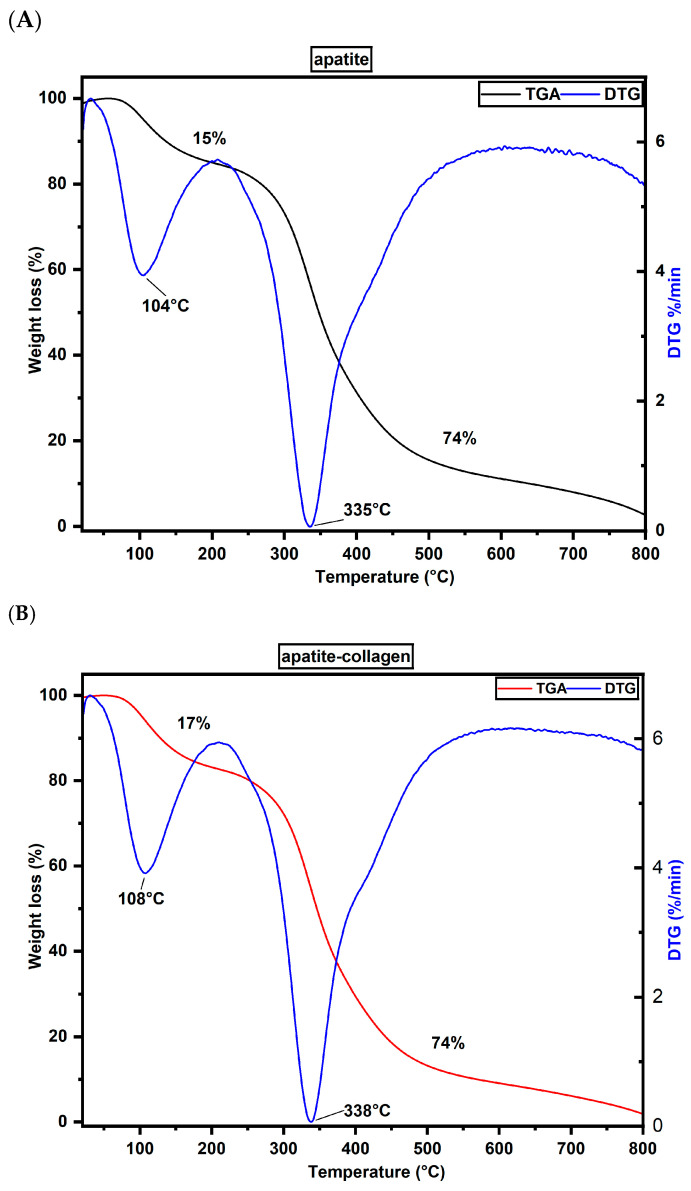
TGA and DTG of apatite and apatite–collagen: (**A**) TGA and DTG of apatite; (**B**) TGA and DTG of apatite–collagen.

Thermogravimetric analysis (TGA) provides information on the thermal stability of materials. Figure 5 shows the thermal degradation by weight of apatite and apatite–collagen post-grafting caused by temperature increase. Figure 5A shows the apatite thermogram, and Figure 5B shows the apatite–collagen, which has two similar degradation states. The first stage of the degradation of apatite occurs between 30 and 210 °C; the second stage is between 210 and 620 °C; and finally, a thermal event occurs between 620 and 800 °C. The first stage of degradation is due to the loss of water adsorbed from the material, with a 15% mass loss [19,23]. The second stage of degradation is related to the breakdown of collagen due to covalent bond breaking, with a mass loss of 74% and a maximum degradation temperature of 335 °C [24,25]. The last thermal event is due to the residual content of the inorganic material, with a mass loss of 8.41% [26,27].

On the other hand, apatite–collagen (Figure 5B) shows a mass loss of 17% in the first degradation stage, attributed to water loss. In the second degradation stage, like apatite, it has a mass loss of 74%; however, in the thermogram derivative (DTG), a maximum degradation temperature of 338 °C is observed for apatite–collagen, which suggests a more remarkable ability to resist higher temperatures before decomposition, thus indicating improved thermal stability [28,29].

**Figure 6:**
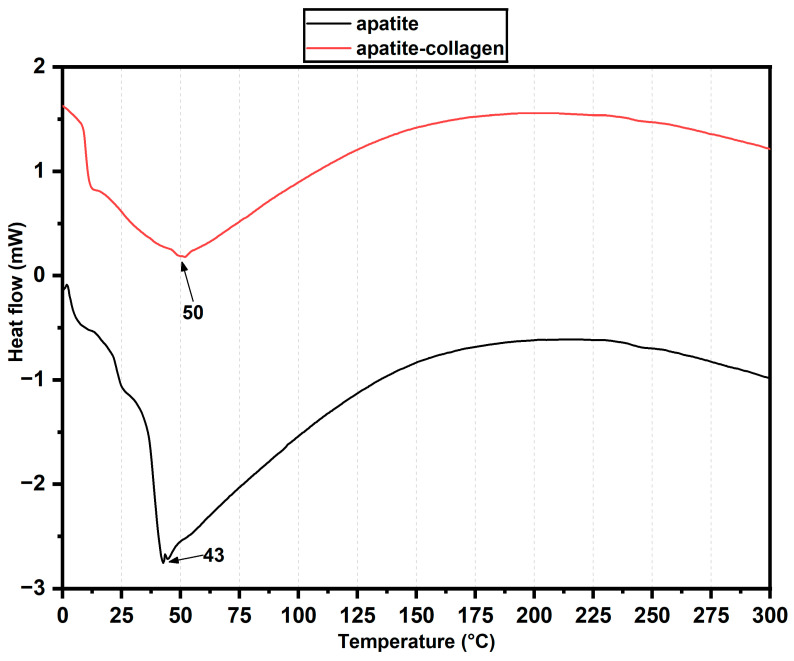
DSC thermograms for apatite (black line) and apatite–collagen (red line).

DSC (differential scanning calorimetry) analysis was used to determine the thermal properties of the compounds. Figure 6 shows the thermograms of the apatite (black line) and apatite–collagen (red line) materials. Endothermic peaks were found at 43 °C and 50 °C for apatite and apatite–collagen. These peaks could be attributed to the dehydration and denaturation of type I collagen, which occurs by the breaking of hydrogen bonds and non-covalent bonds that confer stability to the helical structure of collagen [30,31]. The denaturation temperature of collagen is directly related to thermal stability, with the compound with the highest denaturation temperature being the most stable, which indicates that apatite–collagen, having a higher denaturation temperature, is the most thermally stable compound [29,32]. The absence of other endothermic peaks in the thermogram may be due to thermal events associated with collagen breakdown, which occur at higher temperatures, as observed in the TGA analysis. Additionally, although the presence of peaks corresponding to the inorganic material was expected, these may have shifted to higher temperatures, resulting in their absence in the thermogram [28,33].

### 2.3. Analysis of Changes in the Dimensions of Alveoli Grafted with Apatite and Apatite–Collagen, as Well as in Bone Density

Based on the information obtained from the CT scans, height, width, and density changes were compared for the 30- and 180-day periods studied, as shown in the following table (Table 1).

No statistically significant differences were found between the group with collagen and the group without collagen in any of the variables evaluated (height, width, density, area) or in the differences and percentages of reduction over time.

Concerning the changes observed about time (measurement at 30 and 60 days), Table 2 and Table 3 show the results obtained.

The results showed no significant differences in measurements between the two time points for the group with collagen; however, the density tended to increase.

In the group without collagen, both height and density showed significant increases. In the group without collagen, both height and density showed significant increases.

### 2.4. Microscopic Description of Alveoli Implanted with Apatite and Apatite–Collagen Materials

**Figure 7:**
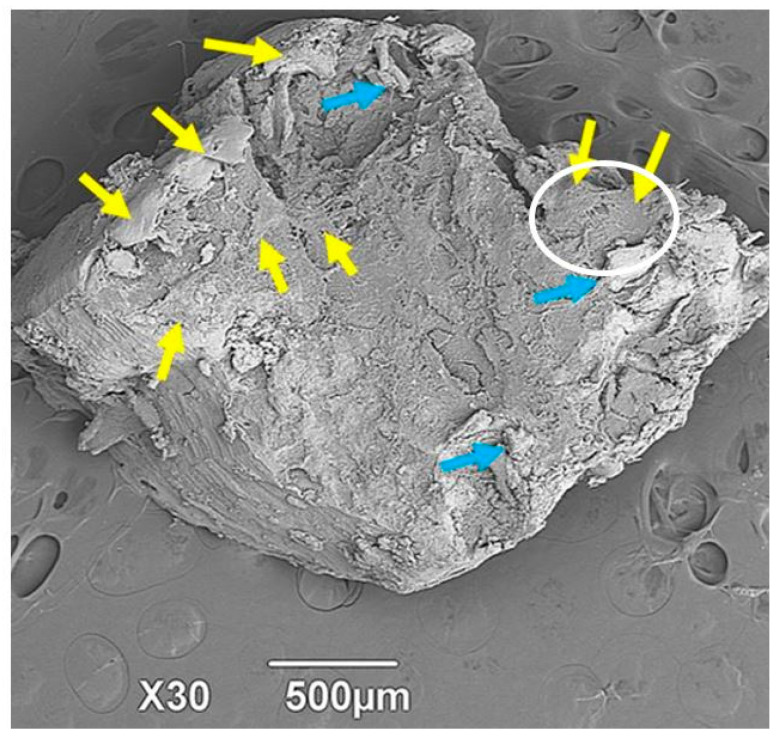
Sample of dental alveoli grafted with apatite material, 6 months after implantation. SEM. Yellow arrows: Osteoblast-like cells. Blue arrows: Deposits of extracellular bone matrix. White circle: Area of histological interest.

Figure 7 corresponds to one of the alveoli grafted with the apatite material. After the healing period, it is observed that the tissue that makes up the biopsy is very compact. The micrograph shows the inner surface of the cylinder covered by cells compatible with osteoblasts, as indicated by the yellow arrows; there are also numerous deposits of extracellular matrix (blue arrows). In this cut, no identifiable particles of the apatite material are visible, and the appearance is of normal bone tissue.

After the teeth are removed, the space left in the dental alveoli is filled with apatite particles without any organic material. This process led to the resorption of the grafted material and its replacement by newly formed tissue, indicating a regeneration process. The presence of osteoblast-like cells near each other and in contact with cytoplasmic prolongations (as seen in the center of the white circle in Figure 7) may indicate that the tissue is undergoing bone formation due to osteoblastic activity. Alternatively, these cells may correspond to bone-covering cells that line the surface of the newly formed tissue.

When the casts of the biopsy material were fractured to observe the internal area of the block, the presence of osteoblast-like bone cells and abundant deposits of extracellular bone matrix were found. Figure 7 shows some images corresponding to samples of some dental alveoli that were grafted with the apatite material.

**Figure 8:**
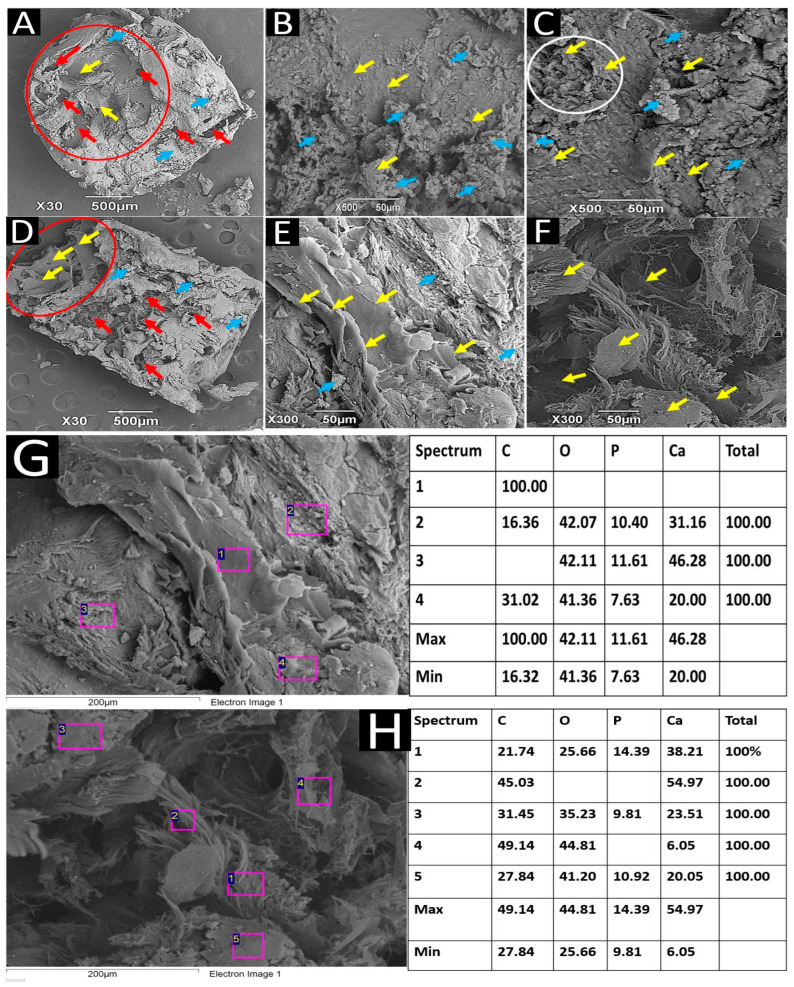
Sample of dental alveoli grafted with apatite material, 6 months after implantation. SEM technique/EDS technique. (**A**) Image of a cross-section of the biopsy. (**B**,**C**) Biopsy surface in cross section with extracellular matrix deposition. (**D**–**F**) Image of a longitudinal section of another biopsy with osteoblast-like cells. (**G**) Spectral analysis of the section shown in (**E**). (**H**) Spectral analysis of the section shown in (**F**)**.** Red circle: Cavity inside the sample. Yellow arrows: Osteoblast-like cells. Blue arrows: Deposits of extracellular bone matrix. Red arrows: Resorption lagoons. White circles: Areas of histological interest. Spectrum 1 to 5: Analysis zones. C: Carbon. O: Oxygen. P: Phosphorus. Ca: Calcium.

In bone remodeling, a balance is established between resorption and the neoformation of the bone matrix. When a bone substitute is incorporated, it must be reabsorbed, and osteoblasts use the resulting space to deposit a new bone matrix through creeping substitution [34]. Figure 8A shows a cross-section of a biopsy. On the surface, there is a large cavity (red circle) that is probably the product of the graft material’s resorption. At the bottom of the cavity, there are several osteoblast-like cells in the process of forming a bone matrix. Towards the periphery, the tissue is more homogeneous and has osteoblast-like structures. In general, the entire surface of the sample has small areas compatible with type II osteoclastic resorption gaps [35], indicated by red arrows, in the resolution process, and numerous deposits of extracellular matrix, indicated by blue arrows.

Figure 8B,C correspond to the exact biopsy, where several osteoblast-like cells related to extracellular matrix deposits are identified. The white circle in Figure 8C shows a group of osteoblast-like cells communicated by cytoplasmic prolongations in the deposition of the extracellular matrix.

Figure 8D–F correspond to a longitudinal section of the biopsy of another dental alveolus. Figure 8D shows a group of osteoblast-like cells at one end, highlighted with yellow arrows. In the center of the sample, the white circle also highlights some osteoblasts producing an extracellular matrix. Figure 8E (at a magnification of 300×) allows us to see a large group of osteoblast-like cells amid extracellular matrix deposits. Figure 8F shows a group of osteoblast-like cells interacting with each other through cytoplasmic prolongations. Fibrillar structures that seem to correspond to collagen fibers are also identified.

SEM-EDS analysis of the structures in Figure 8E,F revealed extracellular bone matrix deposits with abundant Ca and P ions. Since calcium and phosphorus are vital components of hydroxyapatite, the principal mineral in bone tissue, the high concentrations of these elements near a group of cells resembling osteoblasts suggest that the tissue is undergoing mineralization. Figure 8G,H show some of the elemental analyses performed. The maximum percentages observed (46.28% and 54.97% for Ca and 11.61% and 14.39% for P) reflect the accumulation of these minerals, which is characteristic of bone tissue in development or active formation. Additionally, the cells resembling osteoblasts are not yet covered by an extracellular matrix, as shown in the microphotographs, indicating that these cells are very active in synthesizing and depositing the bone matrix, corresponding to the initial phase. 

No loose particles of the graft material corresponding to apatite material were observed in any of the biopsies, indicating successful integration into the newly formed tissue despite slow resorption [8]. In addition, the entire surface was covered by structures compatible with osteoblast-like cells, with abundant extracellular matrix deposits.

**Figure 9:**
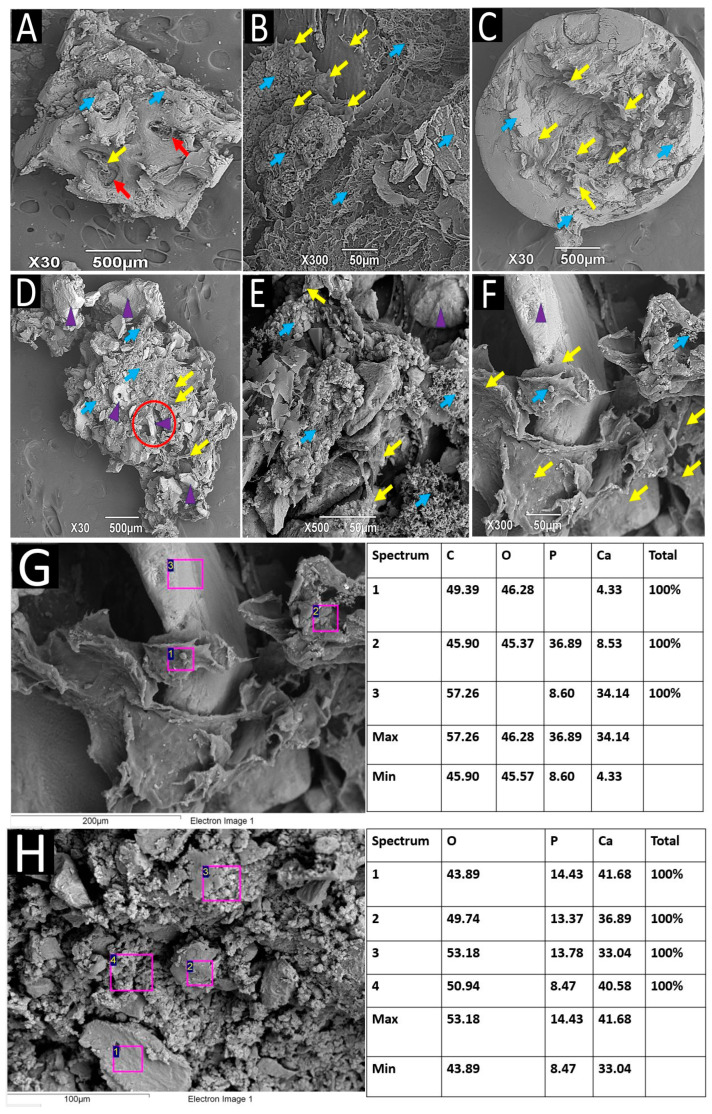
Sample of dental socket grafted with apatite–collagen. SEM technique/SEM technique. (**A**) SEM technique. (**B**) SEM technique. (**C**) SEM technique. (**D**) SEM technique. (**E**) SEM technique (**F**) SEM technique. (**G**) Spectral analysis of sample F, using the EDS technique. (**H**) Spectral analysis of sample F, using the EDS technique. Yellow arrows: Osteoblast-like cells. Blue arrows: Deposits of extracellular bone matrix. Red arrows: Resorption lagoons. Purple arrows: Remnant fragments of grafted material. Spectrum 1 to 4: Analysis zones. C: Carbon. O: Oxygen. P: Phosphorus. Ca: Calcium.

Figure 9 shows the results of grafting with the apatite–collagen material. The microscopic images show similarities with what was observed for the apatite material, such as osteoblast-like cells and abundant extracellular matrix deposits. However, no large cavities are observed that could correspond to areas of resorption of the material, in contrast to what was observed in Figure 9A,D. In studies conducted by other authors, apatite material was found to have a lower number of residual particles when compared to similar materials after three weeks of grafting [36].

The absence of large resorption cavities in the microphotographs of the alveoli grafted with apatite–collagen, together with the presence of abundant extracellular matrix deposits, may indicate a faster resorption and replacement process of the grafted material compared to the other xenograft. Our group reported this when comparing the two materials in a critical-sized intraosseous design in Wistar rats [10].

Figure 9A shows a longitudinal section of a biopsy cylinder, in which it is possible to observe some small cavities compatible with resorption gaps (indicated by red arrows), as well as a small cavity with the presence of cell-like structures (marked by a yellow arrow) and extracellular matrix deposits (marked by blue arrows). Figure 9B (taken at a magnification of 300×) shows a group of osteoblast-like cells depositing an extracellular matrix in the resorption gaps (indicated by yellow arrows) [35], along with numerous extracellular matrix deposits (blue arrows). Figure 9C, from another biopsy, shows a cavity filled with extracellular matrix due to osteoblast-like cell activity (yellow arrows) and some extracellular matrix deposits on the surface.

Figure 9D shows a longitudinal section of a different biopsy from the previous ones to study the sample’s interior. Small resorption lacunae occupied by osteoblast-like cells (yellow arrows) and several extracellular matrix deposits (blue arrows) are observed. A cylindrical structure in a cavity can be discerned in the center of the image, highlighted with a red circle. Figure 9E corresponds to a section of Figure 9D at a magnification of 500×, showing osteoblast-like cells associated with extracellular matrix deposits. Figure 9F shows a magnification of 300× of the area highlighted with the red circle in Figure 9D, where a cylindrical structure, likely a residual particle of the grafted material, is observed, with a group of osteoblast-like cells depositing an extracellular matrix on its surface. This is more clearly evidenced in Figure 9G, corresponding to a spectral analysis of the same area. Spectra No. 1 and No. 2 correspond to the analysis of the material observed on the cells and show the presence of Ca and P in relatively low amounts. In contrast, Spectrum No. 3 corresponds to the residual fragment and shows a very high presence of calcium, as might be expected.

Figure 9H corresponds to the EDS spectral analysis of the same biopsy as Figure 9D. Spectra 1, 2, 3, and 4 were obtained from structures compatible with osteoblast-like cells located in a large extracellular matrix deposit. The results show a significant presence of calcium and phosphorus at high levels.

The two spectral analyses shown in Figure 9G,H correspond to different sectors of the same biopsy. An interesting finding is the close contact between osteoblast-like cells with bovine hydroxyapatite particles, highlighting the osteoconductive effect of the material, which serves as a scaffold for cell colonization. The Ca percentages in Spectrum No. 1 (4.33%) and Spectrum No. 2 (8.53%) are low compared to the Ca content in Spectrum No. 3 (34.14%). For phosphorus, Spectrum No. 2 shows a value of 36.89%, much higher than the 8.6% found in Spectrum No. 3. These low Ca percentages may indicate an early stage of extracellular matrix formation, explaining why the cells are identifiable and show small deposits. In contrast, Figure 9H reveals a large extracellular matrix deposit with some structures compatible with osteoblast-like cells becoming trapped in the matrix. This process, reminiscent of late-stage extracellular bone matrix formation and maturation, involves osteoblasts becoming trapped in the matrix they secrete and transforming into osteocytes. In Spectra 2, 3, and 4, taken from structures compatible with cells, Ca values range from 33.04% to 41.43%, and P percentages range from 8.47% to 14.43%, higher than in other spectra. These results indicate that the material turnover process was not uniform across the samples; some areas show early matrix secretion with minimal mineralization, while others exhibit more advanced mineralization with early bone cell incorporation into the extracellular matrix.

The comparison of the SEM images indicates that the bone remodeling and neoformation process was faster for the apatite–collagen samples. No large cavities are visible, and many cells and ECM deposits are observed. The red circle in Figure 9D shows a small cavity in the process of being reshaped. When looking at this same area at a magnification of 300×, a small particle of the material can be seen supporting a large group of osteoblast-like cells producing an ECM, as can be verified with the spectral analyses in Figure 9G.

The finding of osteoblast-like cells using a remnant particle of material is interesting and indicates an osteoconductive property of the implanted material, which is in agreement with other reports that calcium phosphates and hydroxyapatite possess this quality [37]. This also shows how the remaining particles of bovine apatite are integrated into the newly formed bone tissue, as has also been reported by other authors [36,38].

The EDS shown in Figure 9G allows the mineral contents present in the area to be contrasted. Spectra No. 1 and 2, taken on material deposits on cell surfaces, show moderate amounts of Ca. At the same time, Spectrum No. 1 corresponds to what appears to be an unabsorbed apatite particle with very high levels of Ca, indicating that the matrix being formed is maturing. A review of the EDS analyses presented in Figure 9H shows that all spectra show high percentages of calcium, indicating increased mineralization in this zone.

## 3. Discussion

The difference between apatite and apatite–collagen materials is the presence of collagen fibers. Both have nanopores and mesopores, but collagen fibers have induced an earlier extracellular matrix deposition. When a bone substitute is placed at a regeneration site, it is expected to initially act as an osteoconductor and allow the migration of both bone and blood cells to the graft site (bone grafts, bone substitutes, and orthobiologics), as seems to have occurred for both types of materials, according to the findings reported in Figure 8 and Figure 9. The presence of a barrier material that prevents soft tissue cells from invading the intervention site and allows the remodeling and neoformation process to occur is also required [39,40]. In this work, all alveoli were protected with the same type of barrier, a collagen membrane (InterCollagen^®^ Guide).

Osteoconduction is an essential quality a bone substitute must present in the early stages of the regenerative process. This allows the material, in addition to maintaining the space, to act as a bridge for capillaries to grow and different cells from neighboring areas to arrive [41]. Once the cells colonize the area where the substitute was deposited, the creeping substitution process occurs, whereby the action of the osteoclasts must remove the grafted material, and the osteoblasts simultaneously deposit an extracellular matrix [39]. No osteoclast-like cells were identified in this work, but cavities and resorption lacunae were found where osteoblast-like cells were depositing an extracellular matrix.

The results of this study show that the two materials studied have osteoconductive quality, which is demonstrated in the incorporation of the remnants of material into the newly formed bone and the presence of osteoblast-like cells in intimate contact with apatite particles associated with material deposits. However, the creeping substitution process seems to have occurred differently: in the case of apatite, at 6 months of implantation, large cavities of material in the process of regeneration were observed, while in the case of apatite–collagen, although some cavities were also observed, they were smaller in size. In addition, many structures compatible with resorption gaps were evidenced. In general, the creeping substitution process seems to be happening faster with apatite–collagen, which is consistent with the previous study conducted by our group on cranial bone from Wistar rats [10]. In general, the degradability and resorption of xenografts are considered slow and influenced by the components’ physicochemical characteristics [42]. However, in the case of apatite–collagen, the collagen component is highly degradable, which could affect the overall degradation of the material [43]. There is also the additional advantage that collagen, due to its ability to interact with osteoblasts, has the property of promoting cell differentiation and adhesion [44,45].

In this work, a collagen membrane was used as a barrier in all cases, and tension-free primary closure of the surgical wound was pursued, as this is the standardized protocol at Universidad del Valle for guided bone regeneration techniques. These are also fundamental principles of Guided Regeneration (GBR) techniques.

Primary closure is recommended to prevent wound dehiscence and improve blood supply to the site. In addition, it allows a safe space to be maintained during bone healing [46].

The topic of primary closure is indeed subject to controversy because it can generate more surgical trauma, affect the band of keratinized mucosa, and increase costs [47], as well as post-surgical complications if the wound opens. This can occur when a flap advancement is performed to achieve closure, creating significant tension. However, there are currently different techniques available to achieve primary wound closure without generating tension, such as the split-thickness flap technique and the extension of the incision along the mucogingival line, which are routine procedures in clinical practice and may be used depending on the conditions of the socket and soft tissue [48]. As part of the controversy, in addition to discussing this difficulty, two articles address the loss of keratinized mucosa when healing is not guided toward primary closure. Other articles also mention that using a membrane can minimize ridge loss and reduce the need for additional grafts when placing the implant.

Some consensus meetings, such as the one in Bologna in 2016, endorsed the principle of primary closure, emphasizing stress-free closure and patient and soft tissue analysis [49].

One of the fundamental principles of the guided bone regeneration technique is the use of a membrane. Its benefits have already been clinically and biologically established and include the exclusion of soft tissue cells in the grafted area, the biological stimulation of the bone healing process, the protection of the grafted material, and the optimization of the regeneration space [50,51]. However, other authors recommend leaving it to the discretion of the clinician, depending on the size of the defect to be preserved and its conditions [46].

The standardized graft preservation procedure includes using membranes, the primary closure of tissues without tension, and a waiting time of 4 to 6 months before implant placement. This is because if a particulate graft is placed and the wound is not closed, the material can either come out or become contaminated due to the high number of bacteria in the mouth [11]. It is noted that the function of the membrane is to protect the graft in case of any future failure leading to exposure of the grafted site and to prevent soft tissue cells from colonizing the grafted area, which could lead to the failure of the preservation. This has been established in several consensus meetings, such as the 2016 Bologna meeting, where the use of the membrane, primary closure without tension, and a waiting period of more than 6 months were recommended; the 2022 meeting of the Italian Academy of Osseointegration suggested the membrane to protect the graft and a waiting period of 4 to 6 months before placing dental implants [52]. Additionally, several systematic reviews have reached the same conclusion, including one from 2023 [53].

The statistical analysis of the data from the CT scans on changes in alveoli dimensions and bone density for groups with and without collagen is presented in Table 1, Table 2 and Table 3. Table 1 indicates no statistically significant differences between the groups with and without collagen in the evaluated variables (height, width, and bone density). All *p*-values were more significant than 0.05, suggesting insufficient evidence to reject the null hypothesis of equality between the groups. The variability in the data, as indicated by the standard deviations and interquartile range, may be due to various biological and technical factors affecting the tomographic measurements and bone densities. Additionally, the small sample size (7 subjects with collagen and 11 without collagen) may have hindered the detection of clinically relevant differences.

The dimensions of the alveoli and bone density in collagen samples collected at 30 and 60 days were evaluated, as shown in Table 2. The results indicate no significant differences in height or width between these two periods (*p* > 0.05). Although there was an average increase in bone density from 611.14 Hu to 700.48 Hu, this change was also not statistically significant (*p* = 0.31). These findings suggest dimensional stability and a possible trend of increasing bone density in the collagen group over time. However, it is important to note that the small sample size (n = 14) may have limited the detection of subtle changes.

Table 3 displays descriptive statistics based on the time of sample collection in collagen-free alveoli. Significant changes in width and bone density measurements were observed between the 30- and 60-day periods. Specifically, the mean width increased from 18.73 mm to 19.81 mm (*p* = 0.006), indicating a statistically significant difference. Additionally, bone density showed a notable increase, rising from 526.14 Hu to 721.96 Hu (*p* < 0.001), which is also significant. These findings suggest that the alveoli experienced considerable width and bone density changes over time in the absence of collagen.

In summary, these analyses offer a comprehensive understanding of how collagen conditions and the timing of assessment can impact bone dimensions and densities. This underscores the importance of considering tissue biology and study design when interpreting results.

One of the limitations of this work is the sample size used, which corresponds to 7 alveoli preserved with apatite–collagen and 11 alveoli preserved with apatite. A retrospective sample size determination with a significance level of 0.05 and a statistical power of 80% (accepted standards in biomedical research to ensure statistically robust results) indicated that at least 24 samples per group would be necessary to reach these significance and power levels. However, a review of previous relevant studies, such as one study in 2017, demonstrated that using only 10 alveoli per group allowed for obtaining a power of 80% and a significance level of 0.05 [54]. Similarly, in the work of Sultan et al., *t*-tests with a power of 80% and a *p*-value of 0.05 were used to calculate the sample size, obtaining a result of 10 subjects (5 subjects in each group) [55].

## 4. Materials and Methods

To conduct this pilot clinical study, patients who visited the periodontology graduate clinic at Universidad del Valle in Cali, Colombia, were identified. The study was explained to them, their participation was requested, and their informed consent was obtained.

The inclusion criteria were as follows:

- Patients 22 years of age or older.
- Teeth suitable for extraction.
- Restorative space suitable for an implant-retained restoration.
- Minimum height of 10 mm of alveolar bone to allow implant placement without affecting adjacent vital structures.

Exclusion criteria included the following:

- Periodontal disease.
- Active periapical lesion.
- Systemic diseases of ASA class III.
- Current smokers.
- Inadequate oral hygiene, with a bacterial plaque index greater than 20%.
- Patients who did not cooperate with study follow-up.
- Pregnant women.

Two groups were organized according to the type of grafted material, each composed of 12 randomly assigned participants. Minimally traumatic extraction procedures were performed on the teeth indicated for extraction and replaced with dental implants. Subsequently, alveolar preservation was carried out using two types of xenografts: one composed of bovine apatite and the other of bovine apatite supplemented with collagen. All patients were treated with a collagen membrane (InterCollagen^®^ Guide, Sigma Graft, Fullerton, CA, USA), and tissues were repositioned to achieve primary closure using a 4-0 nylon suture with an anchored mattress technique.

Computed tomography (CT) scans were performed on each alveolus one month and six months after the preservation procedure, and a biopsy of the grafted alveolus was taken. The biopsy, which consisted of a 2 mm diameter and 5 mm long cylinder, was obtained using a trephine-type drill. Each biopsy was segmented into two parts for chemical analysis and scanning electron microscopy (SEM) studies.

The analyses were performed on biopsies taken from 18 patients with grafted alveoli. Biopsies were collected, on average, six months after placement of the grafted material, using trephine drill bits with a diameter of 2 mm and a depth of 5 mm. The material obtained consisted of small cylinders 5 mm long by 2 mm in diameter. Each biopsy was sectioned to obtain two samples. One of the samples was fixed in buffered formaldehyde for further processing and analysis using chemical techniques and scanning electron microscopy.

Of the 24 patients who entered the research, only 18 completed the study. The final groups comprised 11 from the apatite group and 7 from the apatite group with collagen—those who did not finish treatment reported financial difficulties with continuing their dental care. There were no complications during the procedures.

For the analysis plan, bivariate tables were discriminated by type and time of sample collection. The quantitative variables were expressed in mean and standard deviation if the assumption of normality was met and in median and interquartile range in the opposite case. The Shapiro–Wilk test assessed the normality. A MANOVA Multivariate Analysis of Variance was performed for the multivariate analysis, taking the sample without collagen as a reference. A significance level of 5% was considered.

Initially, a sample size of convenience was used, considering the number of patients who requested dental care for alveoli preservation and agreed to participate in the research.

As this was a pilot clinical study, no additional registration was required by the Vice-Rectory for Research and the Ethics Committee of the Universidad del Valle. The ethical protocol for this research was approved by the Health Research Ethics Committee (CEIS) of the Universidad del Valle in Cali, Colombia, through 045-022 of 6 June 2022.

The main possible biases were identified as follows:

Selection bias: In this study, patients were selected from those who attended the Universidad del Valle periodontology graduate clinic, which may limit the generalizability of the results to other contexts or populations. Participants were randomly assigned to one of two treatment groups to minimize selection bias.

Information bias: This bias can occur if there are errors in data collection or interpretation. The study used CT scans and biopsies to evaluate the grafted alveoli. To minimize this bias, standardized procedures for collecting biopsies and CT scans were applied. In addition, each biopsy was divided into two parts for chemical analysis and scanning electron microscopy (SEM) studies, providing a more detailed and robust evaluation. To reduce the impact of bias, an expert researcher performed each procedure: a periodontic specialist for clinical guidelines, an oral implantologist for the interpretation of tomographic images, a biomedical science professional for the interpretation of SEM results, and a chemist for chemical analysis.

Procedural bias: This can occur if procedures are not applied consistently among participants, such as variations in alveoli preservation techniques or xenograft application. To mitigate this, standardized operating protocols were followed.

Temporal bias: To avoid variability during data collection or follow-up, which could affect results, CT scans were performed one month and six months after the procedure, thus minimizing any time-related bias.

Random assignment, standardized procedures, and rigorous inclusion/exclusion criteria were used to control these biases.

### 4.1. Characterization of Post-Implanted Xenografts with Apatite and Apatite–Collagen Material

#### 4.1.1. Fourier Transform Infrared Spectroscopy (FTIR)

Infrared Fourier transform spectroscopy (FTIR) determined the functional groups in the post-implanted xenografts. The characterization was performed on a NicoletTM Summit TM Lite FTIR spectrometer (Thermo Fisher Scientific, Waltham, MA, USA) in a spectral range of 500–4000 cm^−1^ with an average of 32 scans and a resolution of 4 cm^−1^.

#### 4.1.2. Thermal Analysis of Apatite and Apatite–Collagen

After grafting, thermogravimetry was used to determine the water content, organic matter, and inorganic residue of apatite and apatite–collagen. The analyses were conducted on a SETSYS Evolution instrument (Setaran Instrumentation, Caluire, France) using a nitrogen atmosphere at a 50 mL/min flow rate and a 10 °C/min heating ramp from −30 to 800 °C.

On the other hand, to determine the thermal transitions of apatite and apatite–collagen post-graft, differential scanning calorimetry (DSC) analysis was performed using a DSC Standard Cell 2920 (MDSCTM TA Instruments Inc., New Castle, DE, USA) as an instrument. Two cycles of −30–300 °C were performed, with a heating ramp of 10 °C/min under a nitrogen atmosphere and a flow rate of 50 mL/min.

#### 4.1.3. Scanning Electron Microscopy Analysis

The samples were coated with a 15 nm layer of gold with the Cressington 108 auto Sputter Coater (Watford, UK) to improve electron conduction. The analyses were performed with Zeiss EVO MA 10 Scanning Electron Microscope (SEM) equipment, using a secondary electron detector at 20 kV. Finally, elemental analysis was performed using the Model INCAPentaFETx3 instrument (Oxford Instruments, Abingdon, UK) probe for energy-dispersive spectroscopy (EDS). 

#### 4.1.4. Tomographic Study

CT scans were taken with a tomograph (ICAT17-19 Next Generation, Imaging Sciences International LLC, Hatfield, PA, USA) six months after the grafting process was conducted to perform a histomorphometry analysis. The images obtained were used to assess changes in the dimensions of the alveoli. To undertake this, three measurements of the alveolus’s width, height, and bone density were taken using the measurement tool available in the free software Blue Sky Plan, Version 4.12 (Blue Sky Bio, Libertyville, IL, USA). In addition, the calculated areas of the alveoli were determined with the ImageJ program (Java 8 64-bit, Java-ImageJ).

After the grafting period (6 months), a biopsy was taken from the center of the alveolus with a trephine of 2 mm internal diameter and 5 mm in length, obtaining a cylindrical sample of the tissue of 2 mm in diameter by 5 mm in length. This sample was fixed in buffered formaldehyde for 15 days, then dehydrated using 70%, 80%, 90%, and 100% ethanol for 30 min each, and dried in an oven at 37 °C for 5 days.

## 5. Conclusions

FTIR analysis revealed the presence of characteristic clusters of inorganic and organic components of the extracellular bone matrix, including phosphate groups, carbonates, and amides I, II, and III in xenografts recovered from the patients’ alveoli. These groups reflect the good resorption and integration with bone tissue of these xenografts due to the interaction between the apatite and apatite–collagen materials with the bone tissue. The thermogravimetry analysis demonstrated the thermal stability of the materials, showing similar mass losses and maximum degradation temperatures for apatite and apatite–collagen. In addition, the DSC analysis indicated endothermic peaks for both materials, attributed to the denaturation of the triple-helix structure of collagen. However, the material with collagen presented a more significant displacement in the denaturation temperature, with apatite–collagen being the most thermally stable compound.

Based on the results obtained, the evaluated materials demonstrated osteoconductive properties, facilitating hydroxyapatite formation in the preserved alveoli, as evidenced by SEM-EDS. In addition, the apatite–collagen material promotes faster healing due to its osteo-promoting properties, facilitating speedier filling of the spaces.

However, these findings should be interpreted considering the study’s limitations, such as the small sample size and the high data variability. These limitations compromised the ability to detect significant differences between the investigated groups. It is crucial to address these challenges in future study designs to ensure the reliability and validity of the statistical results, which could strengthen the conclusions on the comparative effects of the materials tested on alveolar preservation.

## 6. Limitations of the Study

The study used random assignment, standardized procedures, and strict inclusion/exclusion criteria to minimize biases and enhance the reliability and validity of the results. However, the study’s main limitation is the relatively small sample size.

To address this limitation, it is recommended that a larger-scale study be conducted and supplemented with traditional histological analyses. Additionally, tomographic studies on dental implants that have already been restored would be valuable in comparing potential differences between the materials used and their clinical outcomes.

It is suggested that studies be conducted with a larger sample size, which would allow for complementary histological studies and microcomputed tomography. This would facilitate additional types of analysis and statistical tests, potentially addressing the high variability observed and providing further insights into the behavior of these materials.

Although the comparison with the literature conducted in the retrospective sample determination presents similar or smaller sample sizes than those used in this work, it is crucial to recognize that the impossibility of reaching the proposed sample size may have impacted the study results. Furthermore, it is essential to note that the statistical results showed remarkable variability in the data, possibly attributable to intrinsic biological factors influencing the measurements and results. The small sample size and high data variability limit this study and influence the results obtained. The lack of an adequate sample size is a significant limitation, as it may have reduced the ability of the study to detect fundamental differences between the investigated groups. This observation highlights the importance of carefully considering sample size in the design of future studies to ensure the reliability and validity of the statistical results obtained.

## Figures and Tables

**Table 1:** Descriptive statistics discriminated by xenograft type.

Variable	Type			
	With Collagen	Without Collagen	Total	*p*-Value
**N. n (%)**	**7 (38.89)**	**11 (61.11)**	**18 (100)**	**-**
HEIGHT 1 AVERAGE MM. Mean (sd)	21.51 (7.49)	18.73 (6.98)	19.81 (7.10)	0.44 *
HEIGHT 2 AVERAGE MM. Mean (sd)	21.58 (8.21)	19.81 (7.20)	20.49 (7.42)	0.65 *
DIFFERENCE HEIGHT2—HEIGHT1. Mean (sd))	0.06 (1.84)	1.07 (1.03)	0.68 (1.44)	0.22 *
PERCENTAGE OF HEIGHT REDUCTION. Mean (sd)	−0.46 (8.93)	6.96 (6.34)	4.07 (8.10)	0.08 *
WIDTH 1 AVERAGE MM. Mean (sd)	8.43 (2.16)	9.39 (3.26)	9.02 (2.85)	0.46 *
WIDTH 2 AVERAGE MM. Mean (sd)	8.49 (2.47)	8.55 (3.22)	8.53 (2.87)	0.97 *
DIFFERENCE WIDTH2—WIDTH1. Mean (sd)	0.05 (1.54)	−0.84 (1.40)	−0.49 (1.49)	0.23 *
PERCENTAGE OF WIDTH REDUCTION. Mean (sd)	1.48 (20.48)	−9.07 (14.81)	−4.97 (17.47)	0.26 *
DENSITY 1 AVERAGE Hu. Mean (sd)	611.14 (113.09)	526.14 (199.98)	559.20 (172.79)	0.28 *
DENSITY 2 AVERAGE Hu. Mean (sd)	700.48 (317.79)	721.96 (208.78)	713.61 (247.79)	0.88 *
DIFFERENCE DENSITY2—DENSITY1. Mean (sd)	89.34 (211.90)	195.82 (98.95)	154.41 (156.40)	0.25 *
PERCENTAGE OF DENSITY REDUCTION. Median (IQR)	15.81 (−5.09–33.40)	36.57 (24.81–53.61)	30.28 (9.73–46.82)	0.11 **

* *t*-test. ** Mann–Whitney U test.

**Table 2:** Descriptive statistics discriminated by the time of sample collection in samples with collagen.

Variable	Type: With Collagen			
	30 Days	60 Days	Total	*p*-Value
**N. n (%)**	**7 (50)**	**7 (50)**	**14 (100)**	**-**
AVERAGE HEIGHT MM. Mean (sd)	21.51 (7.49)	21.57 (8.21)	21.54 (7.55)	0.93 *
AVERAGE WIDTH MM. Mean (sd)	8.43 (2.16)	8.49 (2.47)	8.46 (2.22)	0.92 *
AVERAGE DENSITY Hu. Mean (sd)	611.14 (113.09)	700.48 (317.79)	655.81 (233.80)	0.31 *

* t-test paired.

**Table 3:** Descriptive statistics discriminated by the time of sample collection in samples without collagen.

Variable	Type: Without Collagen			
	30 Days	60 Days	Total	*p*-Value
**N. n (%)**	**11 (50)**	**11 (50)**	**22 (100)**	-
AVERAGE WIDTH MM. Mean (sd)	18.73 (6.98)	19.81 (7.20)	19.27 (6.94)	0.006 *
AVERAGE WIDTH MM. Mean (sd)	9.39 (3.26)	8.54 (3.21)	8.97 (3.19)	0.07 *
AVERAGE DENSITY Hu. Mean (sd)	526.14 (199.97)	721.96 (208.78)	624.05 (223.25)	<0.001 *

* t-test paired.

## Data Availability

Data is contained within the article.
